# Delay in seeking health care from community residents during a time with low prevalence of COVID-19: A cross-sectional national survey in China

**DOI:** 10.3389/fpubh.2023.1100715

**Published:** 2023-02-21

**Authors:** Ziyu Wang, Yurong Tang, Yu Cui, Hanwen Guan, Xiaoqian Cui, Yuan Liu, Yanni Liu, Zheng Kang, Qunhong Wu, Yanhua Hao, Chaojie Liu

**Affiliations:** ^1^School of Health Management, Harbin Medical University, Harbin, China; ^2^School of Psychology and Public Health, La Trobe University, Melbourne, VIC, Australia

**Keywords:** COVID-19, delay of care, China, cross-sectional national survey, multivariate logistic regression model

## Abstract

**Background:**

The pandemic of COVID-19 has significant implications on health resources allocation and health care delivery. Patients with non-COVID illness may have to change their care seeking behaviors to mitigate the risk of infections. The research aimed to investigate potential delay of community residents in seeking health care at a time with an overall low prevalence of COVID-19 in China.

**Methods:**

An online survey was conducted in March 2021 on a random sample drawn from the registered survey participants of the survey platform Wenjuanxing. The respondents who reported a need for health care over the past month (*n* = 1,317) were asked to report their health care experiences and concerns. Logistic regression models were established to identify predictors of the delay in seeking health care. The selection of independent variables was guided by the Andersen's service utilization model. All data analyses were performed using SPSS 23.0. A two-sided *p* value of <0.05 was considered as statistically significant.

**Key results:**

About 31.4% of respondents reported delay in seeking health care, with fear of infection (53.5%) as a top reason. Middle (31–59 years) age (AOR = 1.535; 95% CI, 1.132 to 2.246), lower levels of perceived controllability of COVID-19 (AOR = 1.591; 95% CI 1.187 to 2.131), living with chronic conditions (AOR = 2.008; 95% CI 1.544 to 2.611), pregnancy or co-habiting with a pregnant woman (AOR = 2.115; 95% CI 1.154 to 3.874), access to Internet-based medical care (AOR = 2.529; 95% CI 1.960 to 3.265), and higher risk level of the region (AOR = 1.736; 95% CI 1.307 to 2.334) were significant predictors of the delay in seeking health care after adjustment for variations of other variables. Medical consultations (38.7%), emergency treatment (18.2%), and obtainment of medicines (16.5%) were the top three types of delayed care, while eye, nose, and throat diseases (23.2%) and cardiovascular and cerebrovascular diseases (20.8%) were the top two conditions relating to the delayed care. Self-treatment at home was the most likely coping strategy (34.9%), followed by Internet-based medical care (29.2%) and family/friend help (24.0%).

**Conclusions:**

Delay in seeking health care remained at a relatively high level when the number of new COVID-19 cases was low, which may present a serious health risk to the patients, in particular those living with chronic conditions who need continuous medical care. Fear of infection is the top reason for the delay. The delay is also associated with access to Internet-based medical care, living in a high risk region, and perceived low controllability of COVID-19.

## Introduction

The World Health Organization (WHO) declared COVID-19 as a global pandemic in March 2020 (1). As of 1 May 2022, over 500 million confirmed cases of COVID-19 and over six million deaths had been reported worldwide (2). Along with the direct health threats of COVID-19, there have been disruptions to health services (3). History shows that the Ebola outbreak in 2014–2015 created serious interruptions on the availability, uptake, and demand of health care services in Sierra Leone (4).

COVID-19 has put health care services under serious stress all over the world. China had adopted a “dynamic zero-COVID” policy prior to December 2022, which required a quick response from local governments to cut off the chain of community transmission through imposing restrictions and mobilizing available health resources once a new COVID-19 case was identified (5, 6). Community residents could experience additional barriers in seeking health care (7). This has raised serious concerns about the delay or avoidance of health care (8, 9).

Delay or avoidance of medical assessment (10), treatment of bacteremia (11), thrombolysis for stroke (12), and treatment of botulinum toxin (13) has been reported during the outbreak of COVID-19 in various countries. Meanwhile, many people missed the opportunity of early detection of new conditions and failed to manage their existing chronic conditions properly (14–16). In Japan, 5.6% of patients living with chronic conditions reported worse health (17). The state of Victoria in Australia witnessed significant decline in patient visits to hospital emergency departments and the diagnosis of five common cancers dropped by approximately one third, prompting urgent calls for the public to seek timely medical attention (18). Similarly, reduced screening, referrals and presentations for lung and colorectal cancers in the UK also led to a projection of 4.8 and 16.5% increased deaths from the two cancers, respectively (19, 20). A study suggests that efforts to reduce COVID-19-related care avoidance are warranted even in regions with low COVID-19 prevalence (21).

Delay/avoidance of health care can be caused by patient choice and/or as a result of fear of infection and process delays (including disruptions of supply chain) (22, 23). In May 2020, the WHO conducted a global assessment of health services, which showed that service provision had been damagingly impacted by COVID-19 (24–26). In some countries, elective surgeries were suspended to mobilize resources to fight COVID-19 (27). However, most existing studies have attributed delay/avoidance of care during COVID-19 to fear of infection. Meanwhile, fear of losing job, being separated from friends, and falling into financial difficulties have also been acknowledged as the underlined reasons of avoidance of seeking testing for COVID-19 (28).

There is a paucity in the literature documenting the effect of COVID-19 on health care seeking behaviors of consumers. The current study aimed to investigate potential delay of community residents in seeking health care at a time with an overall low prevalence of COVID-19 in China.

## Methods

### Study setting and participants

A cross-sectional online survey was conducted in mainland China. The study protocol was approved by the Research Ethics Committee of Harbin Medical University (IRB code HMUIRB20200004). The survey was anonymous. Participant information sheet was provided and implied informed consent was required from each participant prior to proceeding to the survey.

Study participants were recruited through the online survey platform Wenjuanxing (www.wjx.cn). It has reach to the largest pool of survey participants in mainland China, covering all regions: more than one million questionnaire responses are recorded by Wenjuanxing every day. Eligible participants in this study were the adults over 18 years of age. They were identified randomly through an automation process embedded in the Wenjuanxing sampling services. The identity of the invited participants remained anonymous and unknown to the research team.

### Data collection and retention

Data were collected in March 2021. Only one submission per IP address was allowed. The survey closed when returned questionnaires reached 4,383. To ensure quality of the data, the returned questionnaires containing logical errors (contradictory answers) and those that were completed within 10 min were excluded. Our pilot test showed that at least 10 min would be needed to read through the questionnaire. This resulted in a final sample size of 4,325 (98.75% of the returned questionnaires). Of those, 1,317 (30.45%) reported health problems and intention to seek medical care.

### Study measurements

The questionnaire development was informed by the existing literature (29) and was adapted to the specific context of COVID-19 in line with the relevant guidelines issued by the WHO (30), the National Health Commission and the national CDC in China (31).

Delay in seeking health care was the main interest of the study. Participants were asked to report their self-assessment of health and intention to seek health care over the past month, when the seven-day rolling average of daily new confirmed COVID-19 cases ranged from 7.57 to 56.71 (32). For those who intended to seek health care, their experiences in obtaining the needed care were further investigated through a series of questions, which included whether they ‘delayed care due to concerns related to COVID-19' (1 = “yes”; 0 = “no/unsure”), for what condition (cardiovascular and/or cerebrovascular diseases, digestive diseases, bone diseases; respiratory disease, eye nose throat diseases, diabetes mellitus, tumor, accident and injury, and others), through what service (medical consultation, emergency treatment (care for immediate life-threatening conditions), obtainment of medicines, follow-up examination, hospital admission, surgical procedure, and others), and from which provider (local provincial/municipal public hospitals, primary healthcare network, cross-provincial/municipal public hospitals, private clinics or private hospitals). In addition, they were asked to identify one or more reasons for the delay, if applied, from the following list: fear of infection; discouragement from relatives and friends; difficulties with online appointments; long waiting time in facilities; complex service procedure; facility closure; denied access to facilities; transfer to infection/fever clinics; movement restrictions; community lockdown; complex referral procedure; and others.

The study participants who reported delay in health care were also asked to identify one or more consequences they anticipated (including disrupted medication, slow recovery, complications, missed optimal timing of treatment, deterioration of illness conditions, dissatisfaction with care provision, increased costs, increased mental burden on family, and others) and how they coped with the delay (including use of Internet-based medical services, self-treatment at home, family/friend help, telemedicine, government assistance, and others).

The selection of independent variables was guided by the Andersen's service utilization model (29), which categorizes predictors of health service utilization into predisposing, enabling, and need factors. In this study, gender (male vs. female), age ( ≤ 30, 31–59, ≥60 years), and marital status (married vs. others) were deemed as predisposing factor, while residency (urban vs. rural), risk level of regions (high vs low), educational attainment (with or without a university degree), personal income (≥average (5,000) vs. < 5,000 Yuan per month (33)), health insurance coverage (yes vs. no), pregnancy or co-habitant with a pregnant woman (yes vs. no), and using Internet medical services (yes vs. no) measured enabling factor. In China, more than 50 accumulative active cases of COVID-19 in a municipality over a period of 14 days would be classified as high risk (7).

Need factor was measured by COVID-19 risk perception and chronic conditions (yes vs. no). The measurement of risk perception followed the definition of Bauer from Harvard (34), considering people's cognition, feeling, and comprehension of the risk characteristics, not the actual risk (35). Adams and Smith (36) pointed out that individual risk perception is closely associated the severity and controllability of the risk. Risk perception is a pivotal determinant of care seeking behaviors (37). In this study, a three-dimensional scale was adopted to measure COVID-19 risk perception, covering perceived susceptibility to COVID-19 infection, perceived severity of the consequences of COVID-19, and perceived controllability of COVID-19 outbreaks. Each dimension contains three items rated on a six-point Likert scale ranging from 1 (strongly disagree) to 6 (strongly agree). A summed average score was calculated for each dimension, with 1–3 indicating a low level and 4–6 indicating a high level of risk perception. The risk perception scale has been validated in a previous study (38). Good internal consistency (Cronbach's α = 0.824) and construct validity (GFI = 0.982, AGFI = 0.961, IFI = 0.972, CFI = 0.972, RMSEA = 0.062 in confirmatory factor analysis) of the scale were also evident in this study. Chronic conditions were defined as a general term for the diagnosed diseases with an insidious onset and prolonged course (39), which include cardiovascular diseases, cerebrovascular diseases, diabetes, and others (40).

### Data analysis

The percentage distributions of the study participants with different characteristics were described and compared between those living in high and low risk regions using Chi-square tests. A multivariate logistic regression model was then established to determine the significant predictors of delay in seeking health care after adjustment for variations in other variables. The reasons of delay and perceived consequences were described and ranked in order using percentage distributions.

All data analyses were performed using SPSS 23.0. A two-sided *p* value of < 0.05 was considered as statistically significant.

## Results

### Participant characteristics

Of the 1,317 study participants who reported a need to seek health care, 832 (63.2%) lived in high risk regions over the study period. Over half were female (54.1%), married (54.5%), obtained a university qualification (52.2%), and had no chronic conditions (55.4%) at the time of the survey. The vast majority were younger than 60 years (95.2%), resided in an urban area (67.1%), earned < 5,000 Yuan per month (61.7%), had health insurance coverage (93.5%), and were not pregnant or living with a pregnant woman (95.9%). Although 81.9% of respondents perceived high severity of COVID-19, 70.1% perceived high levels of controllability, and 87.9% perceived low levels of susceptibility. Compared with the respondents from a region with low mobility restrictions, those experiencing high mobility restrictions were older (*p* < 0.001), and were more likely to be married (*p* < 0.001), obtained no university qualifications (*p* = 0.01), earned a low level of income (*p* = 0.012), had no health insurance coverage (*p* < 0.001), lived with chronic conditions (*p* = 0.01), and perceived higher levels of susceptibility (*p* < 0.001) and lower levels of controllability (*p* = 0.012) (Table 1).

**Table 1:** Characteristics of study participants reporting a need to seek health care (*n* = 1,317).

**Characteristics**	**Total**		**High-risk regions (*****n*** = **832)**		**Low-risk regions (*****n*** = **485)**		***p***
	***n***	**%**	***n***	**%**	***n***	**%**	
**Gender**							0.347
Male	605	45.9	374	45.0	231	47.6	
Female	712	54.1	458	55.1	254	52.4	
**Age (Years)**							< 0.001
≤ 30	593	45	301	36.2	292	60.2	
31–59	661	50.2	471	56.6	190	39.2	
≥60	63	4.8	60	7.2	3	0.6	
**Marital status**							< 0.001
Married	718	54.5	491	59.0	227	46.8	
Others	599	45.5	341	41.0	258	53.2	
**Residency**							0.304
Urban	884	67.1	550	66.1	334	68.9	
Rural	433	32.9	282	33.9	151	31.1	
**Educational attainment**							0.010
University	688	52.2	412	49.5	276	56.9	
Without university	629	47.8	420	50.5	209	43.1	
**Monthly personal income (Yuan)**							0.012
High (≥5,000)	504	38.3	297	35.7	207	42.7	
Low (< 5,000)	813	61.7	535	64.3	278	57.3	
**Health insurance coverage**							< 0.001
Yes	1,231	93.5	760	91.4	471	97.1	
No	86	6.5	72	8.7	14	2.9	
**Living with a pregnant woman**							0.263
Yes	54	4.1	38	4.6	16	3.3	
No	1,263	95.9	794	95.4	469	96.7	
**Using Internet medical service**							0.072
Yes	531	40.3	320	38.5	211	43.5	
No	786	59.7	512	61.5	274	56.5	
**Chronic disease**							0.010
Yes	588	44.6	394	47.4	194	40.0	
No	729	55.4	438	52.6	291	60.0	
**Perceived severity of COVID-19**							0.958
High	1,079	81.9	682	82.0	397	81.9	
Low	238	18.1	150	18.0	88	18.1	
**Perceived controllability of COVID-19**							0.012
High	923	70.1	563	67.7	360	74.2	
Low	394	29.9	269	32.3	125	25.8	
**Perceived susceptibility of COVID-19**							< 0.001
High	160	12.1	125	15.0	35	7.2	
Low	1,157	87.9	707	85.0	450	92.78	

### Delay in seeking healthcare services

Overall, 31.4% of the study participants who had a care need experienced delay in seeking health care: 35.8% in those residing in a high risk region compared with 23.7% in those from low risk regions (p < 0.001). However, living in high risk regions (AOR = 1.736 [95% CI 1.307–2.334]) was not the only predictor of delay in seeking health care. An age between 31 and 59 years (AOR = 1.535 [95% CI 1.132–2.246]), lower levels of perceived controllability (AOR = 1.591 [95% CI 1.187–2.131]), living with chronic conditions (AOR = 2.008 [95% CI 1.544–2.611]), pregnancy or co-habitant with a pregnant woman (AOR = 2.115 [95% CI 1.154–3.874]), and access to Internet-based medical services (AOR = 2.529 [95% CI 1.960–3.265]) were also associated with delay in seeking health care according to the results of the multivariate modeling. The associations of delay in seeking health care with middle age, marriage, urban residency, higher personal income, and absence of health insurance coverage became statistically insignificant after adjustment for variations of other variables. The multivariate model explained 19.2% of variance (R^2^) in delay of seeking health care (Table 2).

**Table 2:** Delay in seeking health care in study participants with different characteristics (*n* = 1,317).

		**Delay in seeking health care (*****n*** = **413)**							
**Predictor**	**Respondents reporting a need to seek health care**	***N***	**%**	**Unadjusted OR**	***p***	**Adjusted OR**	**95% Confidence interval**		***p***
**Predisposing factor**									
**Gender**									
Female (Reference)	712	237	33.3						
Male	605	176	29.1	0.822	0.102	0.874	0.677	1.129	0.303
**Age (Years)**									
≤ 30 (Reference)	593	137	23.1						
31–59	661	252	38.1	2.051	0.000	1.535	1.132	2.246	0.007
≥60	63	24	38.1	2.048	0.010	1.268	0.961	1.673	0.093
**Marital status**									
Others (Reference)	599	149	24.9						
Married	718	264	36.8	1.756	< 0.001	1.232	0.907	1.672	0.182
**Enabling factor**									
**Residency**									
Rural (Reference)	433	120	27.7						
Urban	884	293	33.1	1.293	0.046	1.194	0.897	1.589	0.225
**Risk regions**									
Low (Reference)	485	115	23.7						
High	832	298	35.8	1.795	< 0.001	1.736	1.307	2.334	< 0.001
**Educational attainment**									
Without university degree (Reference)	629	189	30.1						
With university degree	688	224	32.6	1.124	0.327	1.308	0.986	1.737	0.063
**Monthly personal income (Yuan)**									
Low (< 5,000) (Reference)	813	237	29.2						
High (≥5,000)	504	176	34.9	1.304	0.028	1.055	0.794	1.401	0.713
**Health insurance coverage**									
Yes (Reference)	1,231	376	30.5						
No	86	37	43.0	1.718	0.017	0.622	0.378	1.025	0.062
**Living with a pregnant woman**									
No (Reference)	1,263	383	30.3						
Yes	54	30	55.6	2.872	< 0.001	2.115	1.154	3.874	0.015
**Using Internet medical service**									
No (Reference)	786	179	22.8						
Yes	531	234	44.1	2.672	< 0.001	2.529	1.960	3.265	< 0.001
**Need factor**									
**Chronic disease**									
No (Reference)	729	171	23.5						
Yes	588	242	41.2	2.282	< 0.001	2.008	1.544	2.611	< 0.001
**Perceived severity of COVID-19**									
Low (Reference)	238	75	31.5						
High	1,079	338	31.3	1.009	0.955	0.565	0.279	1.145	1.113
**Perceived controllability of COVID-19**									
High (Reference)	923	310	33.6						
Low	394	103	26.1	1.429	0.008	1.591	1.187	2.131	0.002
**Perceived susceptibility of COVID-19**									
Low (Reference)	1,157	354	30.6						
High	160	59	36.9	1.325	0.109	1.029	0.750	1.412	0.113

Table 3 shows that the types of care being delayed appeared to be consistent between those from the high and low risk regions, despite some differences in the percentage distributions. Medical consultations (38.7%), emergency treatment (18.2%), and obtainment of medicines (16.5%) were the top three types of delayed care. Eye, nose, and throat diseases (23.2%) and cardiovascular and cerebrovascular diseases (20.8%) were the top two conditions relating to the delayed care, followed by digestive diseases (10.1%) in those living in a region with high risk and bone diseases (12.2%) and respiratory diseases (12.2%) in those living in a region with low risk. Most of the delayed services were planned to be obtained from local provincial/municipal hospitals (68.0%) and primary health care networks (17.2%). Self-treatment at home was the most likely coping strategy (34.9%), followed by Internet-based medical care (29.2%) and family/friend help (24.0%) (Table 3).

**Table 3:** Types of delayed care (*n* = 413).

**Delayed care**	**Total**		**High-risk regions (*****n*** = **298)**		**Low-risk regions (*****n*** = **115)**	
	***n***	**%**	***n***	**%**	***n***	**%**
**Purpose of intended visit**						
Medical consultation	160	38.7	117	39.3	33	28.7
Emergency treatment	75	18.2	52	17.5	23	20.0
Obtainment of medicines	68	16.5	43	14.4	25	21.7
Follow-up examination	50	12.1	40	13.4	10	8.7
Hospital admission	29	7.0	23	7.7	6	5.2
Surgical procedure	21	5.1	13	4.4	8	7.0
Others	10	2.4	10	3.4	10	8.6
**Illness condition to be treated**						
Eye, nose, throat diseases	96	23.2	68	22.8	28	24.4
Cardiovascular and/or cerebrovascular diseases	86	20.8	57	19.1	29	25.2
Digestive diseases	35	8.5	30	10.1	5	4.4
Bone diseases	34	8.2	20	6.7	14	12.2
Diabetes mellitus	33	8.0	26	8.7	7	6.1
Respiratory diseases	30	7.3	16	5.4	14	12.2
Tumor	29	7.0	26	8.7	3	2.6
Accident and injury	19	4.6	12	4.0	7	6.1
Others	51	12.3	43	14.4	8	7.0
**Institution in which care to be sought**						
Local provincial/municipal public hospitals	281	68.0	198	66.4	83	72.2
Primary healthcare network	71	17.2	52	17.5	19	16.5
Cross-provincial/municipal public hospitals	38	9.2	31	10.4	7	6.1
Private clinics or private hospitals	23	5.6	17	5.7	6	5.2
**Coping strategy**						
Self-treatment at home	144	34.9	105	35.2	40	34.8
Internet-based medical services	121	29.2	84	28.2	37	32.2
Family/friend help	99	24.0	74	24.8	26	22.6
Seeking government support	28	6.8	17	5.7	10	8.7
Others	21	5.1	18	6.0	2	1.7

**Figure 1:**
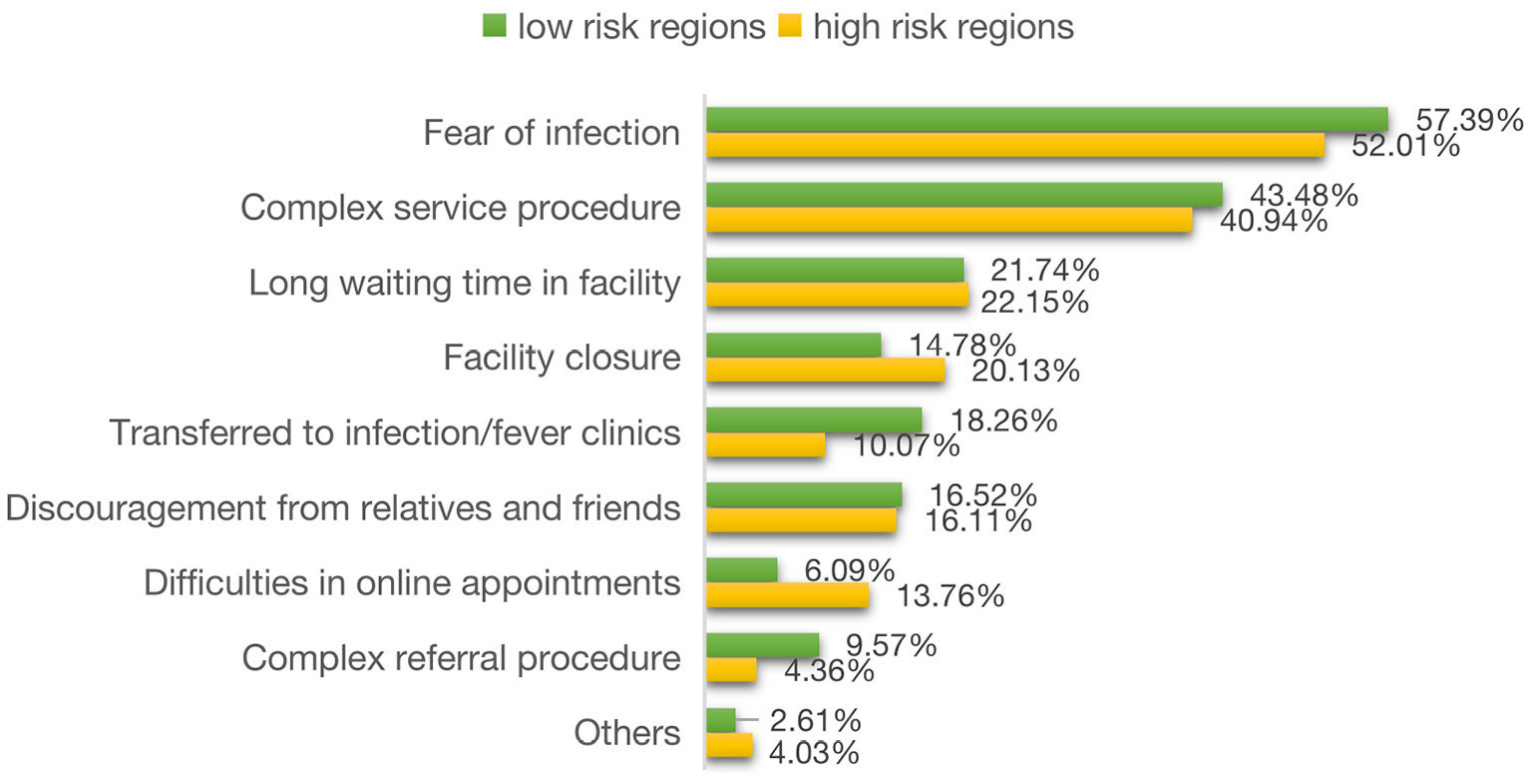
Reasons of delay in seeking hospital care.

Figure 1 shows that the top three reasons of delay in seeking health care were fear of infection (52.01−57.39%), complex service procedure (40.94%~43.48%), and long waiting time in facilities (21.74−22.15%) according to the reports of the respondents from both regions with high and low COVID-19 risk. Of the other reasons, those from the regions with high risk were more likely to report facility closure (20.13 vs. 14.78%), but less likely to report transference to infection/fever clinics (18.26 vs. 10.07%) as a reason of the delay compared with their counterparts from the regions with low risk (Figure 1).

**Figure 2:**
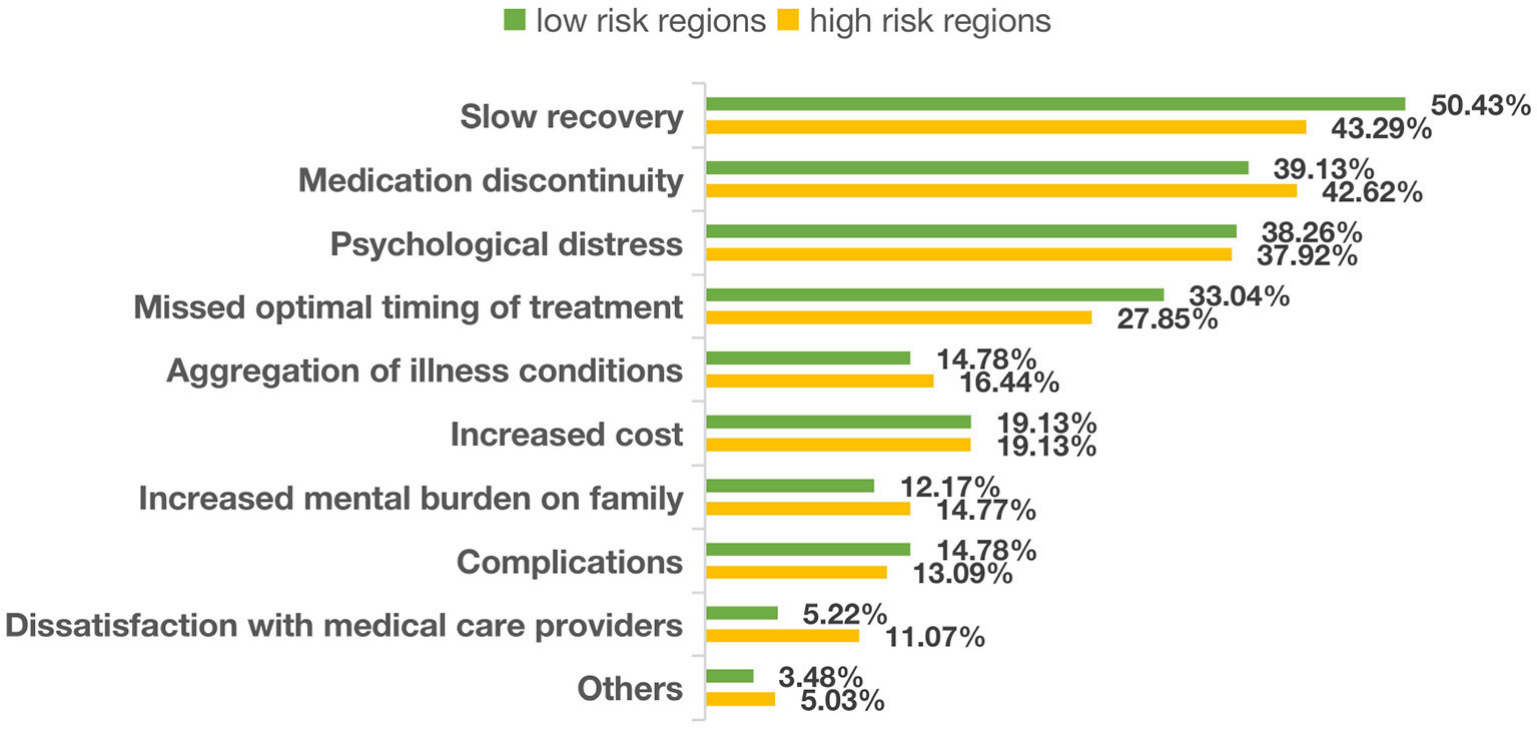
Perceived consequences of delay in seeking hospital care.

Figure 2 shows that the perceived consequences of delayed care followed the same pattern between those living in the regions with high and low risk. Slow recovery (43.29−50.43%) was the top concern, which was followed by disruptions in medication (39.13−42.62%), psychological distress (37.92−38.26%), missed optimal timing of treatment (27.85−33.04%), and deterioration of illness conditions (14.78%−16.44%) (Figure 2).

## Discussion

The COVID-19 pandemic has disrupted healthcare services around the world, which may have serious implications on population health outcomes. Our study shows that 31.4% of patients in mainland China experienced delay in seeking health care over a period with low prevalence of COVID-19. This level of delay is relatively lower compared to those experienced by other countries, whether in the settings with high prevalence of COVID-19 such as the US (65.7%) (41), or in the settings with low prevalence of COVID-19 such as New Zealand (55%) (42). There is emerging evidence indicating serious consequences of delayed health care seeking. A study in a tertiary care center in India showed that 44.7% of pregnancy complications over the period of COVID-19 outbreak were resulted from delay in health care seeking (43).

In our study, eye, nose and throat diseases and cardiovascular/cerebrovascular diseases were reported by respondents as the top two conditions for which needed care was delayed. The delay was system wide, with the majority (68%) occurred with planned care in local provincial/municipal hospitals. A systematic review concludes that COVID-19 has a significant impact on health care seeking behaviors of patients with cardiovascular diseases, causing longer delays between the onset of the symptoms and hospital treatment (44). However, there are limited studies reporting delay in care for eye, nose and throat diseases.

In our study, fear of infection was identified as the major reason for delay in seeking health care, followed by complex services procedure. These findings are consistent with the results of previous studies (45). COVID-19 outbreaks have caused worldwide shortage of health workforce and medicine supplies, transportation difficulties, and even closure of certain medical care services (46). Thaddeus and Maine (47) described three common delays—in seeking care, in reaching the facility, and in receiving adequate treatment. The high contagious nature of the SARS-CoV-2 virus and its relatively high death toll has fueled serious fear of nosocomial infection. Indeed, among the early cases of COVID-19, nearly 41% were suspected to be infected during their hospital visits (48). The risk of nosocomial exposure can trigger psychological panic and avoidance of medical care, especially in the vulnerable populations (49).

Control of the pandemic of COVID-19 has drained tremendous resources that may otherwise be used for other health care services, and led to increased complexity in services procedure. Many hospitals in China started to only accept patients who had an online appointment, despite the challenge of making online appointments by some patients, especially the elderly (50). Normal body temperature was required to get access to non-COVID related treatment, despite a lack of robust evidence to support such practice (51). Additional precaution measures were put into place for hospital services and surgical treatment, including infection risk assessment, nucleic acid testing, and even chest computerized tomography (CT) (52, 53). The increased complexity has added barriers for patients to seek timely care. Similar findings were also reported in other countries. In the United States, for example, patients experienced longer waiting time and the existing racial and socioeconomic inequities in health care were exacerbated by the COVID-19 outbreak (54, 55). Mobility restriction measures, such as travel restriction, suspension of public transport, isolation of infected cases, and quarantine of close contacts are also associated with delay in seeking health care according to the findings of our study and the exiting literature (56, 57). In some countries, governments even imposed nationwide lockdown to contain COVID-19 (58).

The predictors of delay in seeking health care identified in our study cover all of the three categories of factors proposed by Anderson (59): predisposing factor (age), enabling factor (mobility restriction, living with a pregnant woman, using Internet-based medical services), and needs factor (chronic conditions, risk perception). Indeed, delay in health care seeking is a result of balancing act that is shaped by the felt urgency of care need, perceived risk of infection, and self-coping ability under a constraint environment (42). We found that the residents aged between 31 and 59 years are most likely to experience delay in seeking health care after adjustment for variations in other variables. This is consistent with the findings of the studies conducted elsewhere. The potential reasons include economic and functional limitations, while the biological and pathological factors may also contribute to the delay of treatment among 31–59 years old adults (60). We also found that residents living in high risk regions were more likely to report delay in seeking health care, which may be associated with higher levels of mobility restrictions. Meanwhile, it is also interesting to note that pregnancy or living with a pregnant woman and use of Internet-based medical services are associated with delay in seeking health care. This may have reflected the common value and coping strategies adopted by the Chinese people: priority in family protection of the pregnant women and unborn babies and using the Internet-based medical care to minimize risk of infection (61). COVID-19 has triggered a surge of Internet-based medical care (62). However, it is considered as part of self-management in China, which is not considered a complete patient care (63). Delay in seeking health care is more likely to be seen in those with chronic conditions according to the findings of this study and others (64). Empirical evidence shows that patients with chronic conditions are particularly vulnerable to COVID-19. They have a much higher COVID mortality rate than the general population (65), and tend to take extra precaution to avoid health facilities for fear of infection (66). Both COVID-19 and chronic conditions have been proven to be associated with anxiety and depression (67, 68). It is common in the public to see health care facilities as the most dangerous place due to the high risk of nosocomial infection and high death toll of COVID-19 (69, 70). Our study revealed consistently high levels of perceived severity of COVID-19 in the respondents across the regions with high and low risk. However, low levels of perceived susceptibility and high levels of perceived controllability are also evident in this study, which may offer some explanation about the relatively low level of delay in seeking health care in China.

### Limitations

This study adopted a cross-sectional design, which does not allow us to compare the levels of delay in seeking health care before and after the outbreak of COVID-19. No causal conclusions can be established either. The population was not stratified for sampling although a simple random sampling strategy was adopted through the Wenjuanxing platform. We intended to obtain a maximal sample size without calculating the statistical power. The final sample size far exceeds the requirements of a statistical power of 0.8, with an α of 0.05. The final study sample was biased toward those residing in the regions with high risk and those younger than 60 years. The measurement of delay is also subject to recall bias. Attempts to generalize the results to the entire population in China need to be cautious.

## Conclusions

Delay in seeking health care remained at a relatively high level in mainland China (albeit lower than in some other countries) when the prevalence of COVID-19 cases was low: more than 30% patients delayed or avoided needed care. This may present a serious health risk to the patients, in particular those living with chronic conditions who need continuous medical care. Fear of infection and complex service procedures are the major underlying reasons of delay/avoidance of health care, in particular in relation to eye, nose, and throat diseases and cardiovascular and cerebrovascular diseases. Access to Internet-based self-care, restrictions on population movements in high risk regions, and perceived low controllability of COVID-19 are also associated with delay in seeking health care during COVID-19 in China. Although self-treatment at home with support from the Internet-based advices may mitigate some consequences, further studies are needed to unveil the full consequences of delay/avoidance in seeking health care.

## Data availability statement

The raw data supporting the conclusions of this article will be made available by the authors, without undue reservation.

## Ethics statement

The studies involving human participants were reviewed and approved by the Ethics Committee of Harbin Medical University. IRB code is HMUIRB20200004. The patients/participants provided their written informed consent to participate in this study. Written informed consent was obtained from the individual(s) for the publication of any potentially identifiable images or data included in this article.

## Author contributions

YH and CL took overall responsibility for the study design, coordination of the survey, setting up the study framework, and writing. ZW, YT, and YC drafted the manuscript, conducted the survey, and data analyses. XC, HG, YuL, and YaL participated in the literature review and data analyses. ZK and QW participated in the design of the research and revision of the manuscript. All authors contributed to the article and approved the submitted version.
